# Spotlight on prostate cancer: the latest evidence and current controversies

**DOI:** 10.1186/s12916-015-0311-x

**Published:** 2015-03-24

**Authors:** Sigrid Carlsson, Andrew Vickers

**Affiliations:** Department of Surgery (Urology Service), Memorial Sloan Kettering Cancer Center, New York, NY USA; Department of Urology, Institute of Clinical Sciences, Sahlgrenska Academy at University of Göteborg, Göteborg, Sweden; Department of Epidemiology & Biostatistics, Memorial Sloan Kettering Cancer Center, 485 Lexington Avenue, New York, NY 10017 USA

**Keywords:** Prostate cancer, Prevention, Screening, Treatment, Risk stratification

## Abstract

Recent decades have seen dramatic changes in the management of prostate cancer based on novel research findings. Prostate-specific antigen (PSA) screening has been introduced, and then recently modified to include new strategies and biomarkers. Management of advanced disease has been transformed by the rapid introduction of new agents. We have moved from a “one-size-fits-all” approach in prostate cancer management to multidisciplinary strategies tailored to the individual patient and his specific cancer. This editorial marks the launch of the article collection *Spotlight on prostate cancer* (http://www.biomedcentral.com/bmcmed/series/SPR), and here, guest editors Sigrid Carlsson and Andrew Vickers give an overview of the past, present, and future of prostate cancer research and management.

## Editorial

### Introduction

In the nineteenth century, prostate cancer was described as a “very rare disease” [1]. It is now the most common male cancer, and the second or third most common cause of cancer-related death in men in the US and in Europe [2,3]. Our new series, *Spotlight on prostate cancer,* will address a broad range of research and clinical topics related to this common and important public health challenge. We welcome submissions of research articles covering prostate cancer epidemiology and prevention, screening and risk stratification, disease management and new therapies, biomarkers, molecular genomics, and translational studies.

During the past three decades, we have witnessed tremendous shifts in the way that we approach prostate cancer. The introduction of prostate-specific antigen (PSA) screening dramatically altered the presentation of the disease, but we are now moving away from a “one-size-fits-all” approach to new ways of individualizing screening. Better understanding of prostate cancer biology has led to the introduction of several new agents for advanced disease, transforming what had previously been a relatively barren field. Marker research has rapidly developed, with novel genomic markers now identifying the molecular drivers of aggressive disease. We are less aggressive in our approach to low-risk disease, moving away from radical treatment toward an increased use of active surveillance, while becoming more aggressive in attempting to control high-risk tumors. Instead of administering hormonal treatment alone, we also attack the tumor-invaded organs with surgery or kill the cancer cells with radiation.

Are we on the way to solving the quandary of prostate cancer, eloquently expressed by Dr. Willet Whitmore as: “Is cure necessary in those for whom it is possible, and is cure possible in those for whom it is necessary?”. Every approach involves harms and benefits. Every strategy can be debated. The intricacies and challenges of trying our best to understand and manage this disease continue to puzzle and to fascinate us. This new series, *Spotlight on prostate cancer,* will include commentaries as well as debate and opinion pieces.

Although knowledge of the etiology of prostate cancer is still an area of investigation, evidence of nutritional and dietary targets for primary prevention is accumulating. In the review *Nutrition, dietary interventions and prostate cancer: the latest evidence*, Lin and colleagues provide an overview of recent literature on the possible influences of diet and nutrients on prostate cancer outcomes [4].

The controversy surrounding whom to screen, and how best to screen, continues. We have witnessed the rise of screening with PSA. We have observed the rapid and dramatic increased incidence of the disease in the US, followed by a decrease in prostate cancer mortality [2], after the widespread implementation of PSA testing, together with improvements in treatment [5]. We have followed the success of large-scale European screening trials that demonstrate a benefit of screening on prostate cancer mortality [6,7].

Every other year or so we read updated and conflicting guidelines [8], with the United States Preventive Services Task Force (USPSTF), as the outlier, now recommending against the use of routine screening [9].

In the commentary *Prostate-specific antigen based screening: Controversy and guidelines*, Kim and Andriole review the four largest randomized trials of screening and treatment and provide some reasons why these studies yielded apparently conflicting results [10]. Some readers may interpret the results of the screening trials as conflicting; the European ERSPC trial [6] indicating “yes, screening works” and the US-led PLCO trial [11] indicating “no, it does not”. Some could make the same claim about the trials of radical prostatectomy versus watchful waiting; the Scandinavian SPCG-4 trial [12] indicating “yes, radical prostatectomy works” and the US PIVOT trial [13] indicating “no, it does not”.

In our view, however, it is not a question of whether screening and treatment work or do not work. The question is: For which men, and in which situations, might they work, when used appropriately and in a risk-stratified manner?

The settings in which these four studies took place played important roles. While the American population was being heavily screened - essentially changing the question of the PLCO away from an evaluation of the effects of screening versus no screening - the European control population remained largely unscreened, and concomitantly, screening was shown to reduce prostate cancer mortality. Men in the Scandinavian population in SPCG-4 had clinically palpable, higher-risk disease, and surgery was abandoned for men with positive nodes; men in the American population in the PIVOT trial had mainly PSA-detected, lower-risk disease, were older, and surgery was more aggressive.

Owing to the heterogeneity of the disease, risk stratification has become an important aspect of screening, diagnosis, management, and treatment of prostate cancer. With personalized medicine, we attempt to tailor the right treatment, for the right patient, at the right time. Modern management of prostate cancer now includes active surveillance, that is, monitoring the disease and delaying curative intervention until signs of disease progression. Robot-assisted radical prostatectomy is now more commonly performed than open procedures. Our approach to high-risk disease is multidisciplinary, and we continue to make progress in the treatment of advanced prostate cancer with the advent of new drugs for castration-resistant disease, and finding the most effective sequence in which to administer them. Reports on prostate cancer management and treatment are encouraged and will be welcome in *Spotlight on prostate cancer*.
